# The Pseudokinase TRIB1 in Immune Cells and Associated Disorders

**DOI:** 10.3390/cancers14041011

**Published:** 2022-02-17

**Authors:** Richard Danger, Yodit Feseha, Sophie Brouard

**Affiliations:** 1 CHU Nantes, Nantes Université, INSERM, Center for Research in Transplantation and Translational Immunology (CR2TI), UMR 1064, F-44000 Nantes, France; richard.danger@univ-nantes.fr (R.D.); yoditfeseha@gmail.com (Y.F.); 2 LabEx IGO “Immunotherapy, Graft, Oncology”, F-44000 Nantes, France

**Keywords:** *Tribbles* homolog 1, TRIB1, proliferation and differentiation, T cells

## Abstract

**Simple Summary:**

TRIB1 is at the center of major cell signaling pathways. In this review, we describe its role in immune cells and highlight TRIB1 interacting partners which suggests cell-specific functions and that TRIB1 is involved in cellular homeostasis and also in different cancers and immune-related disorders.

**Abstract:**

Research advances in *Tribbles* homolog (TRIB) genes have established the consensus that this protein family plays roles in diverse biological conditions and regulates intracellular signaling networks and several human diseases. In this review, we focus on one member of the family, TRIB1, and its role at the crossroads of immune signaling. TRIB1 directly interacts with transcription factors such as FOXP3 and C/EBPα, with several signaling molecules such as MEK1 and MALT1 and directly acts on key cell signaling pathways such as the MAPK and NF-κB pathways. Altogether, these interactions emphasize that TRIB1 is at the center of major cell signaling pathways while TRIB1 has cell-specific roles, potentially depending on the expressing cells and binding partners. In this review, we describe its roles in immune cells and highlight the interacting partners explaining these functions which suggests TRIB1 as a precise mediator of cellular homeostasis as well as in different cancers and immune-related disorders.

## 1. Introduction

*Tribbles* homolog (TRIB) gene characterization dates back to 1996 when Wilkin et al. identified a member of the Tribbles protein family differentially expressed in the dog thyroid gland [1]. Soon thereafter, the *Drosophila melanogaster Tribbles* gene was identified among genes involved in the regulation of embryonic development [2,3,4]. *Tribbles* was determined to act by negatively regulating the cell cycle regulator *String*/*Cdc25,* supporting its suppression of expression, thus allowing the gastrulation phase to take place and delaying the initiation of mitosis [3,4]. These early studies highlighted essential hallmarks of TRIB protein family members, with specific roles of TRIBs in specific developmental stages [2,3], in modulating specific protein targets [3,5], and having cell-specific roles [6]. Research advances in TRIBs have established the consensus that this protein family plays roles in diverse biological conditions and regulates intracellular signaling networks and multiple human diseases, as reviewed in [7]. TRIB1 roles are guided through interacting partners that confer cell-specific functions, notably in immune cells, the focus of this review.

## 2. Overview of the TRIB1 Gene and Protein

**Figure 1:**
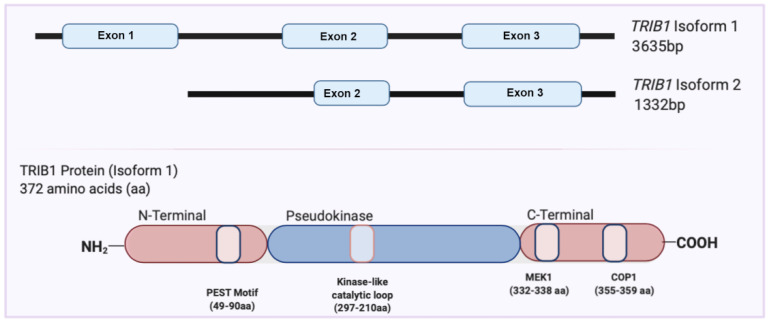
Schematic representation of *TRIB1* isoforms ((**upper**) panel) and TRIB1 protein structure ((**lower**) panel). Created with BioRender.com.

The *TRIB1* gene is located on chromosomes 8q24 and 15 in humans and mice, respectively. The *TRIB1* gene is moderately expressed in various organs and tissues, including the thyroid gland, bone marrow, liver, and lung. *TRIB1*, with three exons, has two validated transcript isoforms: isoform 1 contains all three exons (ENST00000311922), and isoform 2, found in low abundance, consists of exon 2 and exon 3, a shortened 5′UTR (untranslated region), and an additional 5′ coding region not found in isoform 1 (ENST00000520847) (Figure 1) [8].

The TRIB protein family is a unique branch of pseudokinases, a subbranch of the Ca^2+^/calmodulin-activated protein kinase subfamily within the human kinase protein kinome [9,10]. TRIB proteins are characterized based on their ‘kinase-like domain’, which strongly resembles that of the serine-threonine kinases, and their distinct N- and C-terminal regions [11]. The N-terminal segment of Tribbles proteins is commonly observed to be 60–80 amino acid (aa) residues long [11]. The abundance of these aa residues is a key feature within the protein consisting of proline (P), glutamic acid (E), serine (S), and threonine (T) (PEST) sequences. These PEST sequences are involved in controlling the half-life of proteins by regulating their susceptibility to degradation [12,13], which is in agreement with reports showing that Tribbles proteins have high turnover [14,15]. The N-terminus also contains sequences rich in proline, which is characteristic of phosphorylation sites in proteins [11]. Finally, the N-terminus of TRIBs, specifically, TRIB1, has been reported an essential segment involved in the nuclear colocalization of the protein [15].

The TRIBs ‘kinase-like’ domain is characterized by 12 subdomains containing 10–30 aa residues. The kinase-like domain of TRIBs, although similar to kinase domains, lacks the classical motif known to be essential for adenosine triphosphate (ATP) binding that is usually present in kinase proteins [11]. The pseudokinase domain differs in the absence of the aspartic aa in the Asp-Phe-Gly motif, constituting the DFG motif that acts as a ligand for coordination of the Mg2^+^ ion, which is essential for its catalytic activity toward ATP [11]. In agreement with computational analyses, TRIB1 and TRIB3 were reported to not possess any catalytic activity, while TRIB2 was identified to have nucleotide-binding properties with weak kinase activity in vitro [16,17,18].

The C-terminus of TRIB proteins, rich in charged amino acids, has been established to be essential for protein–protein interactions described later [11]. Additionally, a distinct peptide motif in the C-terminus interacts directly with small subsets of cellular E3 ubiquitin ligases, facilitating proteasome-dependent degradation of a network of transcription factors, with their turnover determining the biological attributes of the TRIB proteins [19]. Furthermore, the crystal structure of TRIB1 has been determined by several groups and shows that the protein binding domains in TRIB1 are located mainly in the C-terminus [18,20].

## 3. A Role for TRIB1 in Physiopathology

Our understanding of TRIB1 is consistent with the consensus that it acts as a protein adaptor or scaffold protein, with several studies implying that TRIB1 plays a key role in the regulation of cell differentiation, proliferation, and metabolism while contributing to manifold pathologies (Table 1).

Regarding cancer, as the human *TRIB1* gene is located at a chromosomal locus (8q24.13) near the *MYC* oncogene, it has been associated with multiple cancer types [21]. This 8q24 locus is a well-known amplicon that is amplified in several cancer types; there is strong potential that *TRIB1* is coamplified in this region, which could account for many of the implications of TRIB1 in several cancer types [22]. For example, TRIB1 promotes leukemic transformation [23] and is strongly upregulated in pancreatic cancers, where it supports proliferation and survival [24]. In colorectal cancer, TRIB1 promotes cell migration and invasion by activating the FAK/Src and ERK pathways [25]. However, while *TRIB1* amplification has been demonstrated in prostate cancer [22], such amplification in other cancers remains to be demonstrated; it has been shown in an animal model of AML but not in human AML datasets [22].

TRIB1 has been linked to several immune-mediated diseases that have kindled an interest in studying the role of the gene in several cell types (Table 1). Ashton-Chess et al. (2008) reported that TRIB1 was associated with chronic antibody-mediated rejection (CAMR) in renal transplantation patients in a microarray meta-analysis [26]. This study compared the gene expression profiles of PBMCs and graft biopsies of several graft loss conditions. *TRIB1* was identified as differentially expressed in patients with CAMR in contrast to other histological and clinical diagnoses, both in blood and biopsies. This finding was further validated in a rodent transplant model; overexpression of *TRIB1* was identified in this model, similar to findings in patients [26]. Identifying potential minimally invasive biomarkers such as TRIB1 is essential, as CAMR is a major cause of graft loss more than 1 year after transplantation [27] that is characterized by a progressive decrease in graft function leading to late graft dysfunction and graft loss. In a different gene expression dataset, *TRIB1* was identified as overexpressed in patients on dialysis and those with chronic rejection [28]. Additionally, *TRIB1* expression was found to be increased in B cells from patients with SLE both during clinically inactive disease and in quiescent patients [29]. SLE is a severe and heterogeneous systemic autoimmunity disease that mostly affects women and causes immune-mediated inflammation leading to glomerulonephritis and vasculitis. TRIB1 has also been found to be overexpressed in patients with HIV [30], and *TRIB1* SNPs has been identified to be associated with inflammatory bowel disease (IBD) [31,32] and eczema [33]. From a transcriptomic meta-analysis, we also found that *TRIB1* was overexpressed in colon biopsies from patients with ulcerative colitis (UC) compared to controls, irrespectively of their response to anti-TNFα therapy [34].

The role of *TRIB1* in macrophages has been linked to the development of macrophage-associated diseases, including coronary heart disease. Johnston et al. (2019) recently showed that *Trib1* deficiency in BMDMs led to increased plasma cholesterol and triglyceride levels, resulting in reduced atheroma formation, while *Trib1* transgene expression increased atherogenesis [35]. Thus, TRIB1 may participate in protection against chronic heart disease. Moreover, in metabolic disease, single-nucleotide polymorphisms (SNPs) at the *TRIB1* locus were found to be associated with cholesterol and LDL-C levels [36]. These SNPs were shown to have a significant impact on the levels of triglycerides and LDL, which are associated with CAD; this finding was replicated in multiple ethnic groups [37,38]. In addition, TRIB1 was shown to affect lipid and glucose homeostasis via its well-known molecular mechanism of regulating C/EBPα by leading to its degradation [36,39,40].

Along with TRIB1, the TRIB protein family members TRIB2 and TRIB3 have attracted interest in terms of understanding their functional roles in diseases, mostly cancer and metabolic diseases [22,41]. In contrast to TRIB1, so far, they have been less studied specifically for their role in immune cells [42].

**Table 1:** TRIB1 association in human diseases.

Disease	Description	Reference
Renal transplantation	Increased *TRIB1* expression in PBMCs and biopsies from patients with CAMR	Ashton-Chess et al. 2008 [26]
Human Immunodeficiency Virus (HIV)	Overexpression of TRIB1 in PBMCs from HIV patients	Rome et al. 2020 [30]
Systemic Lupus Erythematosus (SLE)	Increased expression of *TRIB1* in B cells from SLE patients	Garaud et al. 2011; Simoni et al. 2018 [29,43]
Inflammatory Bowel Disease (IBD)	*TRIB1* SNP associated with IBD in patients	Liu et al. 2015; Jostins et al. 2012 [31,32]
Eczema	*TRIB1* SNP associated with eczema	Grosche et al. 2021 [33]
Coronary Artery Disease	*TRIB1* locus association with plasma triglycerides and coronary artery disease	Burkhardt et al. 2010; Douvris et al. 2014 [36,44]
Acute Myeloid Leukemia (AML)	TRIB1 induces inappropriate C/EBPα protein degradation. TRIB1 cooperates with BCL11A which represses several PU.1 target genes	Yokoyama et al. 2011; Yoshida et al. 2013; Yoshino et al. 2021; Sunami et al. 2002 [23,45,46,47]
Multiple Myeloma (MM)	Higher *TRIB1* expression in bone marrow mononuclear cells from MM patients favoring M2 macrophage polarization	Chen et al. 2020 [48]
Hepatocellular Carcinoma (HCC)	TRIB1 promotes (HCC) tumorigenesis and invasiveness via the downregulation of p53 with the possible involvement of the β-catenin signaling pathway	Ye et al. 2017 [49]
Colorectal cancer	*TRIB1* is overexpressed, similarly to MYC. TRIB1 promotes upregulation of MMP-2 through the activation of FAK/Src and ERK pathways	Y. Wang et al. 2017; Briffa et al. 2015; Camps et al. 2009 [25,50,51]
Prostate cancer	*TRIB1* amplification and over-expression are associated with proliferation, cell survival, and metastasis. TRIB1 regulates the GRP78 endoplasmic reticulum chaperone protein, regulating Akt activation	Mashima et al. 2014; Shahrouzi et al. 2020; Moya et al. 2018 [24,52,53]
Non-small cell lung cancer (NSCLC)	Cisplatin treatment resulted in C/EBPβ-dependent increasing of TRIB1 which cooperates with HDAC1 to downregulate p53 activation. TRIB1 may be involved in chemotherapy resistance TRIB1 is modulated by the activation of the PI3K/AKT pathway, specifically by PIK3CA, in lung epithelial cells	L. Wang et al. 2017; De Marco et al. 2017 [54,55]
Glioma	TRIB1 binds to HDAC1 to inhibit p53 expression in glioma cells and participate in radioresistance	Tang et al. 2015 [56]
Several Amplicon 8q24 Associated Cancers	*TRIB1* gene located at the same chromosomal locus 8q24.13 in close proximity to MYC; potentially co-amplified alongside	Röthlisberger et al. 2007 [21].

## 4. Evidence of TRIB1 Function in Immune Cell Subsets

**Figure 2:**
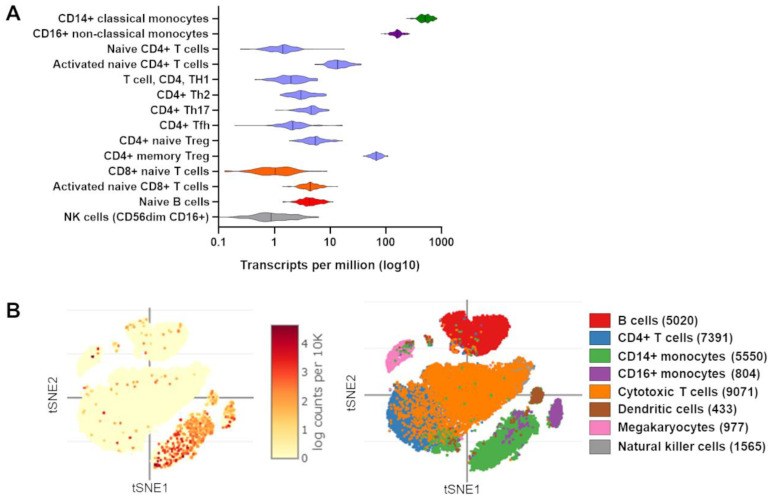
*TRIB1* gene expression in immune cells. (**A**) *TRIB1* gene expression as transcripts per million (TPM, in log10) from bulk RNAseq data from DICE (Database of Immune Cell Expression, Expression quantitative trait loci (eQTLs) and Epigenomics; https://dice-database.org/; accessed on 4 January 2022) are shown [61]. In (**B**), t-SNE clustering of 31,021 PBMCs [62] is represented with each point representing one single cell and with *TRIB1* gene expression colorization and with colors according to cell type in left and right panels, respectively, using the Broad Institute Single Cell Portal (SCP; https://singlecell.broadinstitute.org/single_cell, accessed on 4 January 2022). Predicted cell subtypes are indicated with number of cells in brackets out of the 31,021 PBMCs. For ease of representation, unassigned cells and plasmacytoid dendritic cells are not displayed, corresponding to 46 and 164 cells, respectively.

**Figure 3:**
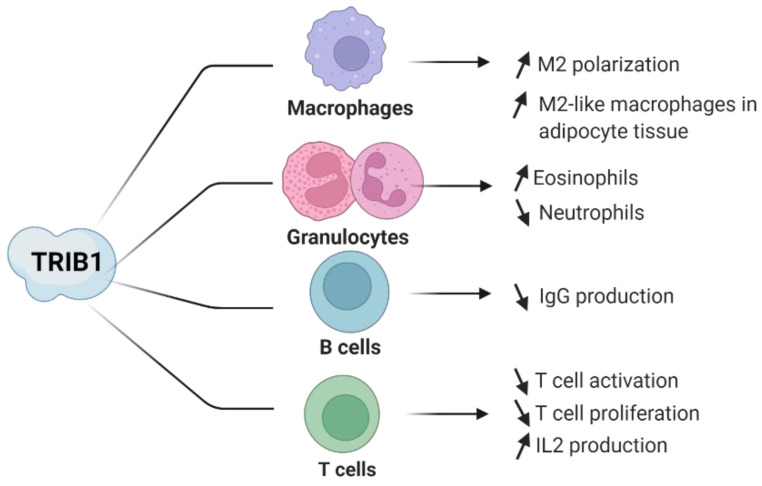
Overview of TRIB1 roles in immune cells. Created with BioRender.com.

Although TRIB1 remains of significant interest in several physiological and pathological conditions, it has also attracted notable attention in terms of understanding its role in immune-mediated diseases and its functional role in several immune cell subsets [57]. In immune cells, *TRIB1* is notably highly expressed in monocytes, CD4^+^ regulatory T cells (Tregs), and activated T cells according to bulk RNA sequencing (RNAseq) of sorting cells (Figure 2A). Single cell RNAseq experiments from PBMCs confirm these results and highlight the higher frequency of TRIB1-expressing monocytes compared to other immune cells (Figure 2B). Previous studies have highlighted the potential role of TRIB1 in B and T lymphocytes, although further elucidation is required [29,30]. Additionally, in CD4^+^ Tregs, progress was previously made by our team, which has incited interest in investigating the role of TRIB1 in Treg cells [58]. Furthermore, studies in myeloblast-derived immune cell subsets of eosinophils, neutrophils, and macrophages have shown a clear molecular role of *TRIB1* in these cells [59,60]. These studies highlight the importance of cell type-specific studies in understanding the functional role of TRIB1 (summarized in Figure 3).

### 4.1. TRIB1 in Macrophages

TRIB1 has been reported by several groups to notably affect macrophages in terms of the polarization toward proinflammatory M1 and anti-inflammatory M2 macrophages [22,61,63]. In vitro, polarization assays of bone marrow-derived macrophages (BMDMs) showed increases of 10-fold and ~5.6-fold in *Trib1* expression in M1 macrophages treated with LPS and interferon-γ (IFNγ) and in M2 macrophages treated with IL-4 within 1 h of stimulation, respectively [64].

The S. Akira group was able to generate mice with global *Trib1* deficiency [65], although other groups, including our group and the E. Kiss-Toth group, experienced lethality issues with full-body *Trib1* deficiency [22]. The S. Akira group stated that these mice showed only slight growth retardation with a reduced body weight until 2–3 weeks after birth. They initially showed that TRIB1 negatively regulates the expression of the NF-IL6 protein, also called C/EBPβ, and is involved in TLR-mediated signaling in peritoneal macrophages [65]. In vivo, BMDMs from these mice with global *Trib1* deficiency showed impaired macrophage polarization with a lower M2 frequency; decreased expression of M2-related genes such as *Cd206*, *Fizz1*, and *Arg1*; and decreased levels of the M2-associated cytokines transforming growth factor β (TGFβ) and IL-10. Even M1 polarized macrophages from *Trib1*-deficient BMDMs showed a lower level of M2-associated genes and cytokine secretion [64]. Arndt et al. also reported that *Trib1* potentially acts on Janus kinase (JAK3) and JAK1, whose protein levels were significantly lower in *Trib1*-deficient macrophages, as their respective targets, signal transducer and activator of transcription 3 (STAT3) and STAT6, exhibited decreased levels of phosphorylation in M1 and M2 macrophages, respectively [64].

In addition, Satoh and colleagues found decreased levels of M2-like macrophages in the spleen, distinguished by their F4/80^+^ MAC1^+^ phenotype and expression of M2-associated genes such *Trc1, Arg1*, and *Fizz1* [59]. *Trib1*-deficient mice with cotransfer of CD45.1 wild-type (WT) and CD45.2 *Trib1*-deficient bone marrow cells showed impaired macrophage development. This accounted for the defect in macrophage differentiation during the developmental stage in the bone marrow. The molecular mechanism was found to be dependent on recruitment of the COP1 E3-ubiquitin ligase by TRIB1, which mediated the degradation of C/EBPα, an essential transcription factor for macrophage differentiation. Furthermore, the increase in C/EBPα expression was suppressed with expression of the *Trib1* transcript in Trib1-deficient bone marrow cells, similar to the molecular mechanism of *Trib1* in eosinophils [59]. Satoh et al. also demonstrated that the role of TRIB1 in M2 differentiation extends to adipose tissue, as the population of M2-like macrophages in adipose tissue was decreased, and high-fat diet feeding resulted in high glucose, insulin, cholesterol, and triglyceride levels in *trib1*-deficient mice [59].

Thus, in macrophages, the functional role of TRIB1 in differentiation has been highlighted, with an important role in M2 differentiation. The effect of TRIB1 seems to be facilitated via its ability to bind to E3 ubiquitin ligase proteins to regulate the C/EBPα ubiquitination level. This role of TRIB1 in macrophage differentiation has been implied and has generated interest in its role in diseases such as atherosclerosis [35].

### 4.2. TRIB1 in CD4^+^ T Lymphocytes

The role of TRIB1 in the total CD4^+^ T cell population was investigated by Dugast et al., who showed a defect in T cell proliferation in total mouse CD4^+^ T cells transduced with a lentiviral vector for *TRIB1* overexpression and activated with α-CD3/α-CD28 [58]. Under *TRIB1* overexpression, cells were able to undergo primary proliferation, while further proliferation was significantly lower than that of cells transduced with the control lentiviral vector [58]. Rome et al. recently confirmed that TRIB1 restrained T cell proliferation using T cells from T cell-specific *Trib1*-deficient mice (*CD4-Cre Trib1*^Flox/Flox^ mice) [30].

Further involvement of TRIB1 in CD4^+^ T cell biology was highlighted in a study investigating the role of TRIB1 in the Jurkat T cell model [63]. Stimulation with phorbol myristate acetate (PMA) and ionomycin induced a significant increase in the *TRIB1* expression level, while the *TRIB2* and *TRIB3* levels were not altered. Similarly, increased *TRIB1* expression was observed following stimulation with only PMA. Via CRISPR/Cas9-directed knockout (KO) of *TRIB1* in Jurkat cells, *TRIB1* was identified to reduce interleukin (IL)-2 production in Jurkat cells [63]. Using an IL-2 promoter luciferase reporter construct, Miyajima et al. found that *TRIB1* regulated IL-2 transcriptional activity in Jurkat cells [63]. Cotransfection of TRIB1 and nuclear factor activator T cell 2 (NFAT2) plasmids led to further enhancement of IL-2 production. In addition to regulating IL-2 production in an NFAT2-dependent manner, TRIB1 was shown to directly interact with histone deacetylase-1 (HDAC1) in a coimmunoprecipitation (CoIP) assay. Miyajima et al. reported that the direct interaction of TRIB1 with HDAC1 could result in disruption of the NFAT-HDAC1 transcriptional complex, leading to enhancement of gene expression—in this case, increasing IL-2 activity [63].

Using a model of chronic viral infection with the chronic lymphocytic choriomeningitis virus (LCMV) clone 13, Rome et al. found an expansion of CD4 effector (CD44^+^ CD62L^−^) and CD8^+^ gp33+ cells and KLRG1^+^ short-lived effector-like T cells in absence of *Trib1* [30]. These expanded CD8^+^ KLRG1^+^ cells exhibited a distinct transcriptomic profile from both progenitor and terminal exhausted CD8 T cell subsets. Interestingly, they confirmed the interaction of TRIB1 with MALT1, also found in a murine B cell line (CH12), and demonstrated that TRIB1 interaction destabilized the CARMA1/Bcl10/MALT1 (CBM) complex which participated in TCR signaling [29,66].

TRIB1 is thus involved in CD4^+^ T cell biology, potentially involved in a negative feedback loop in which TCR activation increases *TRIB1* expression which would restrain T cell activation and proliferation by destabilizing the CBM complex. However, its definite role is not fully understood and while IL-2 is recognized as necessary for T cell survival and proliferation [67], upon activation, TRIB1 enhances IL-2 production [63], which seems to contradict its role in negatively regulating the rate of T cell proliferation [58].

### 4.3. TRIB1 in Regulatory T Cells

Several reports have elucidated that *TRIB1* is highly expressed in CD4^+^ CD25^+^ CD127^−^ Tregs compared with their conventional T cell (Tconv) counterparts in both mice and humans [60,68,69]. Additionally, *TRIB1* is rapidly upregulated in Tregs upon T cell receptor (TCR) stimulation, similar to the response of the transcription factor forkhead box P3 (FOXP3), a master regulator of CD4^+^ Tregs, but *TRIB1* expression decreases after a few hours, in contrast to that of *FOXP3*, which is maintained at a lower but stable level for longer periods [58,70]. The high correlation of *TRIB1* mRNA expression with *FOXP3* mRNA and protein expression suggests that these molecules are coregulated in Tregs and participate in shared pathways [58,68]. Indeed, using a protein complementation assay (PCA), Dugast E. et al. showed that the TRIB1 and FOXP3 proteins directly interact in the nucleus [58].

Two genome-wide FOXP3 ChIP-Seq experiments highlighted FOXP3 binding to the *TRIB1* genomic region both in humans and mice [69,71]. This potentially accounts for the Treg-specific transcription regulation of *TRIB1.* The TRIB1–FOXP3 interaction was further stressed by the finding of increased *Trib1* expression in murine nonregulatory T cells induced to express *Foxp3* [72]. In addition, TRIB1 may be part of the adapted tissue-specific Treg transcriptome; for example, in visceral adipose tissue-derived Tregs, *Trib1* was found to be upregulated, in contrast to spleen Tregs, and its expression was found to be inversely regulated in cells expressing a mutant peroxisome proliferator-activated receptor gamma (*Pparγ*) construct, in contrast to *Pparγ*-expressing Tregs.

Furthermore, the binding of TRIB1 with MALT1 may also play a role in Treg biology as the CBM complex regulates Tregs activation state and suppressive function [73], although no alterations of the Treg compartment have been found in a murine model of chronic viral infection with T cell-specific *Trib1* deletion [30].

Altogether, these studies indicate that TRIB1 is highly regulated in Tregs and interacts with the transcription factor FOXP3, potentially affecting FOXP3 expression or function. However, the functional role of TRIB1 in Tregs has not been evaluated yet.

### 4.4. TRIB1 in B Lymphocytes

In contrast to its expression pattern in CD4^+^ T cells, TRIB1 has been shown to be more highly expressed in resting B cells than in activated B cells [26]. Simoni et al. reported overexpression of *TRIB1* in transcriptomic analysis of peripheral B cells in patients with inactive SLE [29]. This finding was further validated in a second cohort of patients with inactive SLE using qRT–PCR. In vivo, *Trib1* overexpression in the B cell-specific Mb1 (CD79a) Cre model had no impact on the B cell phenotype in the spleen [29]. Under physiological conditions, *Trib1* overexpression led to a decreased serum immunoglobulin (Ig)G1 level, while a decrease in anti-double stranded DNA (anti-dsDNA) IgM production was observed in a model of SLE induced by an injection of lipopolysaccharide (LPS) from *Salmonella typhimurium*. In accordance with these findings, in vitro, a decreased level of IgG1 was observed in the supernatant of splenic B cells treated with LPS and IL-4, which induced IgG1 class switching, although no significant difference in the frequency of IgG1-producing cells was observed. Despite this decrease in IgG1 secretion, no alterations in B cell activation, cell viability, or plasma cell differentiation were observed [29]. Molecular pathway analysis showed decreased levels of phosphorylated ERK upon B cell receptor stimulation, and several protein binding partners of TRIB1, including CD72 and JAK3, were identified using mass spectrometry in murine B cell lines. The involvement of the kinase JAK3 in IL-4 receptor signaling could be associated with the decreased IgG1 level after stimulation with LPS and IL-4 [29].

### 4.5. TRIB1 in Eosinophils and Neutrophils

Considerable research interest in *TRIB1* has focused on its role in the development and progression of acute myeloid leukemia and Down syndrome-related megakaryocytic leukemia [23,74,75]. TRIB1 exerts differential regulatory effects at different stages of granulocyte development, performing a key balancing act in the differentiation and maintenance of both eosinophils and neutrophils [59,60]; that is, TRIB1 acts to promote eosinophil differentiation over neutrophil differentiation. This finding was identified in germline *Trib1* KO mice, which exhibited decreased eosinophil numbers and increased neutrophil numbers [59]. *Trib1* deficiency leads to increased commitment to neutrophil differentiation while blocking early eosinophil commitment, thus favoring myeloid progenitors over neutrophils [60]. In concordance with this observation, Mack et al. showed that *Trib1* deletion in postembryonic hematopoietic cells increased the neutrophil number in the spleen, blood, lung, and colon. Interestingly, in ex vivo eosinophil differentiation, *Trib1* KO results in eosinophils with increased expression of Ly6G, a specific marker of neutrophils, indicating that the absence of *Trib1* favors neutrophil differentiation [60]. Additionally, the existence of these Ly6G+ eosinophils showed that *Trib1* KO results in Ly6G^+^ eosinophils with the inability to suppress neutrophilic characteristics and functions. This study demonstrated that *Trib1* affects eosinophil commitment, differentiation, and function. The molecular mechanism of TRIB1 in eosinophil/neutrophil differentiation is regulated by altering the C/EBPα degradation level, as *Trib1* KO leads to higher amounts of C/EBPα protein, and C/EBPα KO reverses the effect of *Trib1* KO in mice [60].

## 5. TRIB1 Cell Signaling and Protein Binding Partners

**Figure 4:**
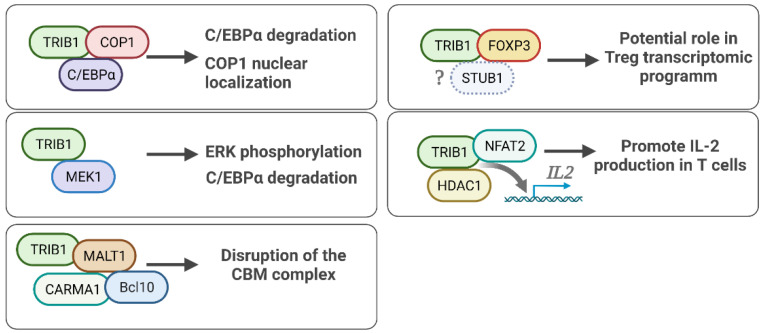
Schematic representation of main and immune-related protein interactions of TRIB1. Created with BioRender.com.

As mentioned earlier, TRIB1 functions depend on interacting partners and may explain some of its cell-specific roles. Recently, a characterization of TRIB1 interactome has been performed by affinity purification mass spectrometry (AP-MS) in human embryonic kidney HEK293T and breast cancer MCF7 cell lines, exhibiting known interactors, such as COP1, and new interactors [76]. Interestingly, TRIB1 was found to interact with itself, suggesting homodimerization as well as heterodimerization with TRIB2. These data suggest a common hallmark of TRIB1 interactome, shared by HEK293T and MCF7 cells, additional to cell- and/or environment-dependent interacting partners which needs more investigation. Thereafter, we focus on main and immune-related protein interactions of TRIB1, displayed in Figure 4.

### 5.1. TRIB1-FOXP3 Protein Interaction

As mentioned above, *Dugast* et al. identified the direct interaction of TRIB1 and FOXP3 using a protein complementation assay with plasmid constructs coding for TRIB1 and FOXP3 and containing complementary segments encoding the GFP [58]. While this construct yielded a strong signal, similar to that of the known TRIB1–MEK1 interaction, this interaction was no longer significant when the TRIB1 N-terminal domain was deleted, while deletion of the C-terminal domain did not result in a significant difference in the binding between TRIB1 and FOXP3. This was further validated using coimmunoprecipitation (CoIP) of FOXP3-TRIB1 from freshly isolated human Tregs. The role of this interaction has not yet been elucidated yet.

### 5.2. TRIB1-COP1 Regulation of the C/EBPα Protein

TRIB1 has been shown to directly interact with COP1, which is an E3 ubiquitin ligase [66]. The TRIB1–COP1 interaction has been shown to facilitate the degradation of the transcription factor C/EBPα, as TRIB1 recruits the COP1 ubiquitin ligase complex to C/EBPα, leading to protein degradation of C/EBPα via ubiquitination [45]. Furthermore, by determining the crystal structure of TRIB1, Murphy et al. evidenced that the C-terminal domain of TRIB1 binds with COP1, forming the ubiquitin ligase complex, which binds to its substrate C/EBPα [18]. As mentioned above, this functional role of TRIB1 has been the key association of TRIB1 in regulating appropriate myeloid cell differentiation, and consequently, dysregulation of this molecular mechanism is understood to play a role in the development of acute myeloid leukemia [45].

Moreover, in addition to the action of TRIB1 in recruiting the E3 ligase COP1 to its substrate C/EBPα, thereby regulating the C/EBPα protein level, Kung et al. also showed that TRIB1 leads to nuclear localization of COP1 [77]. Kung et al. found that in the presence of TRIB1, COP1 nuclear localization was augmented, while in the absence of TRIB1, accumulation of COP1 in the cytoplasm was observed. The TRIB1–COP1 interaction favored nuclear localization of COP1 by directly competing with CRM1-dependent nuclear export [77].

TRIB1, as well as TRIB3, was recently found to interact with the E3 ubiquitin ligases STIP1 and STUB1, expanding the involvement of TRIB1 in protein degradation. Furthermore, STUB1 has been found to mediate the ubiquitination and degradation of FOXP3 in Tregs potentially reinforcing the link of TRIB1 and FOXP3 described above [78].

### 5.3. TRIB1-MEK1 in ERK/MAPK Signaling

The mitogen-activated protein kinase (MAPK) pathway is a highly conserved and essential cellular signaling pathway regulating the processes of cell proliferation, cell differentiation, and cell death [79,80,81]. In murine bone marrow cells, *Trib1* overexpression was found to lead to enhanced and prolonged phosphorylation of ERK, which is linked to suppression of apoptosis [23]. TRIB1 has been shown to bind to MAPKK proteins, including MEK1, MKK4, and MKK7 [82], and this was later shown specifically by its ability to bind MEK1 [23]. Yokoyama et al. revealed this interaction using mutant constructs in HeLa cells, showing that the TRIB1-MEK1 interaction site is at the border between the kinase-like domain and C-terminal region of TRIB1 [23]. This motif is highly conserved between the three mammalian Tribbles proteins [11], and mutants that lack the entire motif, as well as a tryptophan mutant, lose their binding ability [23]. Additionally, this study demonstrated that TRIB1 promotes C/EBPα downregulation through enhancement of MEK1/ERK activity, in addition to its role in regulating C/EBPα degradation through COP1 binding.

Furthermore, the interaction of TRIB1 and MEK1 is abolished when the pseudocatalytic loop of the MEK1-binding domain of TRIB1 is deleted [39]. The MEK1 C-terminal region contains an interaction domain, with the C-terminal deletion mutant of MEK1 showing less binding activity toward TRIB1. However, the role of TRIB1 in the MEK/MAPK signaling pathway is cell-type dependent, either leading to cellular activation and often proliferation or inducing apoptosis [83].

### 5.4. TRIB1 Implication in NF/KB Pathway Activity

Ostertag et al. found that TRIB1 is involved in the proinflammatory cytokine pathway in white adipose tissue (WAT) [84]. *Trib1* KO in WAT was associated with increased expression of genes coding for proinflammatory factors, such as IL-6, IL-1B, interferon-β, and TNFα. A ChIP assay of TRIB1 in 3T3-L1 preadipocyte cells showed direct recruitment of TRIB1 to the promoters of cytokine genes (such as IL-6, TNFα, and IL-1B) in the nucleus and particular recruitment to nuclear factor kappa B (NF-κB)/RELA recognition motif-containing promoter regions [84].

In breast cancer cell lines, TRIB1 expression was identified to regulate the cell cycle by acting upstream of the cell cycle regulator NF-κB [85]. *TRIB1* knockdown inhibited the activity of the NF-κB-responsive promoter, and *TRIB1* overexpression led to an increase in NF-κB promoter activity [85].

### 5.5. Other TRIB1 Protein Binding Partners

Downregulation of *Trib1* expression by shRNA was shown to promote RAR transcriptional activity and result in enhanced expression of endogenous RAR target genes [86]. Moreover, the results of immunostaining and in vitro binding assays strongly suggested that TRIB1 directly binds to RARα in the nucleus. TRIB1 was identified as a negative regulator of RARα. This unique feature of the interaction of TRIB1 with RAR/RXR suggests the existence of previously unknown mechanisms of nuclear receptor-mediated transcriptional repression [86].

Other proteins are likely to bind to TRIB1, as shown in a mass spectrometry analyses, notably by Simoni et al. which identified 236 protein partners in a B cell line (CH12) including MALT1 [29,66]. Another study in CD4^+^ T cells has reported MALT1 and TRIB1 as protein binding partners [30]. The N terminal part of TRIB1 is needed for MALT1 binding as mutation of five residues between amino acid residues 83 and 89 abrogated this binding. TRIB1 inhibited the CBM complex formation supporting the role of TRIB1 in T cell function. Finally, the interaction of TRIB1 with HDAC1 has been reported in cancer cell lines, notably to downregulate p53 activation [54,56] and to enhance IL-2 expression [63].

## 6. Regulation of TRIB1

According to its expression profile in immune cells (Figure 2), TRIB1 is considered to be highly regulated, in accordance with previous studies showing that TRIB1 has considerable instability at the messenger RNA (mRNA) and protein levels. This tight regulation may partially explain different roles of TRIB1 depending on expressing cells.

### 6.1. Transcriptional Regulation of TRIB1

With an mRNA half-life of less than one hour, *TRIB1* is considered among the genes with the shortest mRNA half-life [87]. Co-expression analysis showed that these mRNAs with short half-lives were enriched among genes with regulatory functions, such as transcription factors [87]. Further experiments using inhibitors targeting the elongation and termination stages of mRNA translation showed a 2.5-fold increase in the *TRIB1* expression level. This suggested that the instability of *TRIB1* mRNA is due to the increased activity at the transcriptional level [15].

In addition, several microRNAs (miRNAs)—short noncoding RNAs regulating expression of target genes—have been shown to repress *TRIB1* expression. Using several prediction tools, Nisepo et al. highlighted 1237 miRNAs predicted to target the 3′UTR of *TRIB1* and only 35 were predicted with high confidence and by at least three of these tools [22]. From these 35 miRNAs, miR-101-3p and miR-132-3p were described as direct regulators of *TRIB1* expression in human macrophages and prostate cancer cell lines, respectively. Interestingly, the modulation of these miRNAs alters the inflammatory profile of these cells, including an increase in IL-8 production in macrophages. Among these 35 predicted miRNAs, only miR-23a has previously been shown to directly target the 3′UTR of *TRIB1*, leading to the upregulation of p53 and the induction of miR-23a expression in a possible feedback loop in hepatocellular carcinoma (HCC) [49]. In addition, the miRNAs miR-224 and miR-513b-5p have been shown to act as tumor suppressor by downregulating *TRIB1* expression in prostate cancer and retinoblastoma cells, respectively [22,88]. It was also reported that miR-98-5p could repress *Trib1* expression while this miRNA has been shown to be increased in a mouse model of inflammatory bowel disease (IBD). Its knockdown decreased the expression of genes related to M1 macrophages and enhanced the expression of those related to M2 macrophage and improved IBD symptoms in this mouse model [22]. Therefore, the miR-98-5p effect could be mediated through *Trib1*. Thus, *TRIB1* expression seems tightly controlled by miRNAs, potentially according to the cell type considered.

Recently, the transcription factor cMYC has also been evidenced to bind two regions in the *TRIB1* promoter and, thereby, to induce *TRIB1* expression [22]. The C/EBPβ transcription factor also regulates *TRIB1* expression, notably in anaplastic large cell lymphoma and cisplatin-treated non-small cell lung cancer (NSCLC) [54,89]. As described before, FOXP3 has also been shown to directly interact with the genomic locus of *TRIB1* by chromatin immunoprecipitation (ChIP) both in human and murine CD4^+^ CD25^+^ Tregs [69,71]. In addition to the strong correlation of *TRIB1* and *FOXP3* expression [58,68], these findings strongly indicate a regulation of *TRIB1* by FOXP3 in Tregs.

### 6.2. Posttranslational Modification of TRIB1

TRIB1 has also been reported to be unstable at the protein level, as shown by Souberyrand et al. [15]. This study examined the sensitivity of the TRIB1 protein to alterations in translational and transcriptional activity using cell lines, showing that inhibiting translation using cycloheximide (CHX) resulted in a significant reduction in the TRIB1 protein level (>70%), while inhibiting transcription using ACTD resulted in a 30–40% decrease in its protein level. Time-lapse analysis of the protein half-life in CHX-treated cells showed a half-life of approximately 90 min [15].

Furthermore, an increased TRIB1 protein level was found in the presence of proteasome inhibitors, indicating that the TRIB1 level is controlled by proteasome activity. However, proteasome inhibition did not fully account for the CHX-mediated downregulation of TRIB1, thereby indicating that its instability was not determined solely by the proteasome [15]. Altogether, these studies present TRIB1 as a highly regulated molecule, both at the RNA and protein levels, with notable co-expression with regulatory transcription factors [87], highlighting the functional role of TRIB1 in cooperation with transcription factors. This agrees with the finding that TRIB1 either regulates or interacts with transcription factors.

### 6.3. Cellular Colocalization of TRIB1

The cellular localization of TRIB1 has been reported as both the nucleus and cytoplasm [15,77,90,91]. Even with the same cell lines, different subcellular localizations were observed suggesting that cellular environment impacts TRIB1 localization. For example, while TRIB1 was found equally distributed between the nuclear and cytosolic fractions by Western blot in MCF7 and BT474 breast cancer cell lines [90], TRIB1 was mainly found in the cytoplasm of MCF7 cells in a recent report using confocal microscopy [76].

*TRIB1* transcript mapping to identify a nuclear localization sequence predicted a sequence between amino acids 33 and 51 in the N-terminus of TRIB1 that plays a role in the nuclear localization of TRIB1 [15]. Expression of a flag-tagged TRIB1 construct without the N-terminal residues indeed led to pan-cellular redistribution of TRIB1, confirming the mapping analysis prediction of the residues required for the preferential nuclear redistribution of TRIB1 [15]. This is in agreement with the initial confocal microscopy analysis of the TRIB1 protein fused to green fluorescent protein (GFP) and transfected into HeLa cells, which showed intracellular localization of the protein in the nucleus [91]. Mutation of the N-terminal region of TRIB1 abolished its nuclear localization, while C-terminal deletion resulted in only a reduction in its nuclear localization [91].

Altogether, these findings indicate that TRIB1 appears to be a highly regulated molecule at both the temporal and spatial levels. The spatial regulation advocates either for dual roles of TRIB1 in the cytoplasm and nucleus depending on its present protein partner or for a function of TRIB1 in protein cellular localization by restricting the nuclear entry of its protein binding partner. This is emphasized by recent works by Kung et al., who demonstrated that TRIB1 regulates the nuclear localization of constitutive photomorphogenesis protein-1 (COP1) [77].

## 7. Conclusions

In summary, TRIB1 has been shown to directly interact with master transcription factors such as FOXP3 and C/EBPα, with several signaling molecules such as MEK1 and MALT1 and directly act on key cell signaling pathways such as the MAPK and NF-κB pathways. Altogether, these interactions emphasize that TRIB1 is at the center of major cell signaling pathways, suggesting a role as a precise mediator of cellular homeostasis as well as different cancers and immune disorders. Thus, TRIB1 appears as a potential therapeutic target for various cancers, immune and metabolic diseases. As an example, the use of berberine has recently been shown to enhance *Trib1* expression and reduce diabetic nephropathy in a mouse model [22]. However, depending on expressing cells and binding partners, TRIB1 functions were found to be variable and further comprehension is needed, notably comparing TRIB1 interactome and functions between cell types [76]. Studies highlighting TRIB1 structural conformation and binding partners are providing insights that would help to design high affinity small molecules able to prevent or modulate TRIB1 interactions [19]. Waiting for these molecules and their use in clinic, TRIB1 expression remains a promising molecule to be used as a biomarker in various disorders.
